# Dexmedetomidine Protects Cardiomyocytes against Hypoxia/Reoxygenation Injury by Suppressing TLR4-MyD88-NF-*κ*B Signaling

**DOI:** 10.1155/2017/1674613

**Published:** 2017-11-22

**Authors:** Jin-meng Gao, Xiao-wen Meng, Juan Zhang, Wei-rong Chen, Fan Xia, Ke Peng, Fu-hai Ji

**Affiliations:** ^1^Department of Anesthesiology, First Affiliated Hospital of Soochow University, Suzhou 215006, China; ^2^Department of Anesthesiology, Children's Hospital of Soochow University, Suzhou 215003, China

## Abstract

**Objective:**

We previously reported that dexmedetomidine (DEX) offers cardioprotection against ischemia/reperfusion injury in rats. Here, we evaluated the role of toll-like receptors 4- (TLR4-) myeloid differentiation primary response 88- (MyD88-) nuclear factor-kappa B (NF-*κ*B) signaling in DEX-mediated protection of cardiomyocytes using* in vitro* models of hypoxia/reoxygenation (H/R).

**Methods:**

The experiments were carried out in H9C2 cells and in primary neonatal rat cardiomyocytes. Cells pretreated with vehicle or DEX were exposed to hypoxia for 1 h followed by reoxygenation for 12 h. We analyzed cell viability and lactate dehydrogenase (LDH) activity and measured tumor necrosis factor-*α* (TNF-*α*), interleukin-6 (IL-6), and IL-1*β* mRNA levels, TLR4, MyD88, and nuclear NF-*κ*B p65 protein expression and NF-*κ*B p65 nuclear localization. TLR4 knock-down by TLR4 siRNA transfection and overexpression by TLR4 DNA transfection were used to further confirm our findings.

**Results:**

DEX protected against H/R-induced cell damage and inflammation, as evidenced by increased cell survival rates, decreased LDH activity, and decreased TNF-*α*, IL-6, and IL-1*β* mRNA levels, as well as TLR4 and NF-*κ*B protein expression. TLR4 knock-down partially prevented cell damage following H/R injury, while overexpression of TLR4 abolished the DEX-mediated protective effects.

**Conclusions:**

DEX pretreatment protects rat cardiomyocytes against H/R injury. This effect is partly mediated by TLR4 suppression via TLR4-MyD88-NF-*κ*B signaling.

## 1. Introduction

Cardiac reperfusion is a critical factor that determines prognosis after myocardial ischemia but also leads to further tissue damage and can even increase infarct size. Myocardial ischemia/reperfusion (I/R) injury is a complex pathophysiological process involving a variety of factors and signaling pathways, including oxygen free radicals, calcium overload, inflammation, and apoptosis [1]. Of these, the inflammatory response is a major cause of I/R-induced tissue injury [2].

Toll-like receptor 4 (TLR4), a pattern recognition receptor, is expressed in cells from the myeloid lineage, as well as in cardiomyocytes and microvascular endothelial cells [3]. A previous study reported that TLR4 promotes cardiac dysfunction following myocardial ischemia by activating nuclear factor-*κ*B- (NF-*κ*B-) dependent apoptosis and increasing expression of proinflammatory cytokines [4]. Other studies showed that myocardial injury and inflammation were limited in TLR4-deficient mice after I/R [5, 6] and in* in vitro* TLR4 knock-down in cardiomyocytes [7].

Dexmedetomidine (DEX) is a highly selective *α*_2_-adrenergic receptor agonist that is commonly used in the clinic as a sedative and anesthetic. Clinical evidence has suggested that DEX preconditioning could improve outcomes in patients after cardiac and noncardiac surgeries [8, 9]. Animal studies also showed that DEX preconditioning exerts cardioprotective effects in both* in vivo* and* ex vivo* models [10, 11]. In addition, DEX was reported to inhibit the inflammatory response by suppressing the TLR4-NF-*κ*B pathway in lung and liver tissues [12, 13]. Consistent with the above findings, our recent* in vivo* and* ex vivo* experiments showed that DEX preconditioning alleviated I/R-induced myocardial injury, which was associated with inhibition of inflammatory responses and downregulation of high mobility group box 1- (HMGB1-) TLR4-MyD88-NF-*κ*B signaling [14, 15].

To date, the role of the TLR4 signaling pathway in DEX-mediated cardioprotection has not been fully explored. To further understand the involvement of TLR4 signaling in DEX-mediated cardioprotection against I/R injury, we performed studies in an* in vitro* hypoxia/reoxygenation (H/R) model using the H9C2 cardiac cell line as well as primary cultured rat neonatal cardiomyocytes. We hypothesized that DEX preconditioning protects cardiomyocytes against H/R injury through downregulating TLR4-MyD88-NF-*κ*B signaling.

## 2. Methods

### 2.1. Animals

Neonatal rats (1-2 days old) were provided by the Experimental Animal Centre of Soochow University, Suzhou, China. All animals were treated in accordance with the National Institutes of Health Guide for the Care and Use of Laboratory Animals (NIH publications number 80-23, revised in 1996). The protocol was approved by the Ethics Committee for Animal Experimentation of Soochow University.

### 2.2. Cell Culture

The rat H9C2 cardiomyocyte cell line was obtained from the Shanghai Cell Bank of the Chinese Academy of Sciences. The cells were cultured in high-glucose Dulbecco's modified Eagle's medium (DMEM) (H30243.01, HyClone, USA) containing 10% fetal bovine serum (FBS) (16000-044, Gibco, USA) in an incubator with 5% CO_2_/95% air at 37°C. The culture media were replaced every day and the cells were subcultured for experimental procedures at 80–90% confluence.

Primary rat neonatal cardiomyocytes were prepared as previously described [16, 17]. Briefly, hearts were harvested and placed in ice cold phosphate-buffered saline (PBS). The ventricles were cut into 1–3 mm^3^ pieces and digested in 0.1% collagenase type II (V900892, Sigma, USA) at 37°C for 5 min. The digestion was repeated five times. The supernatants from all digestions were centrifuged (10 min, 1,500x rpm) and finally resuspended in DMEM-F12 (C11330500ET, Gibco) containing 15% FBS. The differential wall adhesion method was used to separate fibroblasts from cardiomyocytes. Resuspended cells were cultured in 5% CO_2_ at 37°C for 2 h, and then the nonadherent cells were extracted and counted with a hemocytometer. Cells in the culture medium were transferred to plates at an appropriate density for subsequent experiments. We added 0.1 mM 5-bromodeoxyuridine (5-BrdU) (B5002, Sigma, St. Louis, USA) to the medium to inhibit fibroblast growth.

### 2.3. H/R Injury Model

The* in vitro* H/R model was established by adding sodium hydrosulfite (Na_2_S_2_O_4_, 71699, Sigma) to the cultured cells. Na_2_S_2_O_4_ was previously reported to induce hypoxia in both H9C2 and primary rat neonatal cardiomyocytes [18–21]. Na_2_S_2_O_4_ removes oxygen from the culture medium without causing any damage to cell membranes, and reoxygenation can be achieved by replacing the medium. The cells were treated with 4 mM Na_2_S_2_O_4_ at 37°C in 5% CO_2_ for 1 h, and then the culture media were replaced with normal media for an additional 12 h to generate a reoxygenated condition.

### 2.4. Experimental Protocols

The experiments were carried out in H9C2 and primary neonatal rat cardiomyocytes. For DEX preconditioning, DEX was added to the culture media 1 h before hypoxia.

(1) To determine the optimal concentration of DEX and investigate whether DEX preconditioning attenuates H/R injury, cells were divided into three groups: group C (control), group H/R, and group D + H/R (DEX pretreatment). In the D + H/R group, cells were treated with 0.1, 1, and 10 *μ*M of DEX for 1 h before hypoxia.

(2) To investigate the effects of TLR4 knock-down by TLR4 siRNA transfection on cardiomyocytes under H/R injury, cells were divided into four groups: group C (control), group H/R, group D + H/R (DEX pretreatment), and group TLR4siRNA + H/R (TLR4 siRNA transfection). In the D + H/R group, cells were incubated with 1 *μ*M DEX for 1 h before hypoxia. In the TLR4siRNA + H/R group, cells were transfected with siRNA using Lipofectamine 2000 (siRNA: Lipofectamine 2000 = 20 nM: 1 *μ*L; Genepharma, Shanghai, China) 24 h before hypoxia.

(3) To investigate whether DEX preconditioning attenuates H/R injury by directly suppressing TLR4 gene expression, cells were divided into four groups: group H/R, group D + H/R (DEX pretreatment), group TLR4DNA + D + H/R (TLR4 DNA transfection), and group CONDNA + D + H/R (control DNA transfection). Before pretreatment with 1 *μ*M DEX, TLR4 DNA/pEX-2 or control DNA/pEX-2 was transfected into the cells using Lipofectamine 2000 (DNA: Lipofectamine 2000 = 1 *μ*g: 2 *μ*L; Genepharma) 24 h before hypoxia.

### 2.5. Cell Viability and Lactate Dehydrogenase (LDH) Assays

Cell viability was measured using the 3-[4,5-dimethylthiazol-2-yl]-2,5-diphenyltetrazolium bromide (MTT) assay, and cell injury was measured using a lactate dehydrogenase (LDH) activity assay. Briefly, cells were seeded in 96-well plates at a density of 1 × 10^4^ cells/well. After inducing H/R, the MTT solution (1 : 10, 0793, Amresco, USA) was added to each well and incubated for 4 h at 37°C. The media were removed and cells were dissolved in dimethyl sulfoxide (DMSO) (V900090, Sigma). The absorbance was measured at 490 nm with a microplate reader (MD, SpectreMax 190). A reduction in optical density reflects a decrease in cell viability. LDH activity was measured using a LDH reagent (C0017, Beyotime, China) at the absorbance of 570 nm according to the manufacturer's protocol.

### 2.6. Western Blot

TLR4, MyD88, and nuclear NF-*κ*B p65 protein expression were detected by Western blot analysis as previously described [22]. The protein concentration was determined using a bicinchoninic acid reagent kit (BCA, P0010, Beyotime). After electrophoresis, proteins were transferred to a polyvinyldifluride (PVDF) membrane at 200 mA for 2 h at 4°C. The blots were probed with the following antibodies at 4°C overnight: mouse anti-rat TLR4 (1 : 100; Santa Cruz Biotechnology; sc-293072), rabbit anti-rat NF-*κ*B p65 (1 : 1000, Abcam, ab7970), mouse anti-rat glyceraldehyde phosphate dehydrogenase (GAPDH) (1 : 2000, MultiSciences, 70-Mab5465-040), rabbit anti-rat MyD88 (1 : 200, Abcam, ab2064), and rabbit anti-rat lamin B (1 : 1000, MultiSciences, 70-ab36361-050). Blots were then incubated with a secondary antibody, goat anti-mouse HRP (1 : 5000, MultiSciences, 70-GAM0072) or goat anti-rabbit HRP (1 : 5000, MultiSciences, lk-gar0072), for 2 h at room temperature. Immunoreactive bands were visualized using an enhanced chemiluminescence kit (ECL, p10100, New Cell Molecular Biotech, China).

### 2.7. Quantitative RT-PCR

Total RNA from cells was extracted with Trizol reagent (Invitrogen, Carlsbad, USA) according to the manufacturer's instructions, and RNA quantity was measured by OD260/OD280. We used the 5x All-in-One RT MasterMix (Applied Biological Materials, Richmond, British Columbia, Canada) for reverse transcription of 1 *μ*g of total RNA as a template. Quantitative real-time PCR was conducted using EvaGreen qPCR MasterMix (Applied Biological Materials) in 10 *μ*L reaction volumes in 96-microwell plates. Relative transcript abundance was determined using the LightCycler 480 software (Roche, Switzerland) according to the 2^−ΔΔCt^ method. *β*-Actin amplification signals were employed as internal controls. Three replicates were performed per sample.

### 2.8. Immunofluorescence (IF) Staining

The cells were seeded into 24-well chambers at a density of 1 × 10^6^ cells/well. After reoxygenation, the cells were washed with PBS, fixed with 4% paraformaldehyde, permeabilized with 1% Triton X-100, and blocked in 3% FBS for 1 h. The cells were then incubated with a primary antibody, rabbit anti-rat NF-*κ*B p65 (1 : 500, Abcam, ab7970), in 1% FBS at 4°C overnight, followed by another incubation with fluorescein isothiocyanate goat anti-rabbit (1 : 500, Abcam, ab150080) for 2 h. The cells were washed and stained with 4,6-diamino-2-phenyl indole (DAPI) for 5 min at room temperature. Immunostained sections were visualized with fluorescent microscopy (Olympus, BX60, Japan).

### 2.9. Statistical Analyses

All data are expressed as mean value ± standard error of the mean (SEM), and one-way analysis of variance (ANOVA) followed by Tukey's test was performed using SPSS 22.0 statistical software (IBM SPSS, Chicago, IL, USA). *P* < 0.05 was considered statistically significant.

## 3. Results

### 3.1. DEX Pretreatment Attenuates Cell Damage and Inflammation in Cardiomyocytes Exposed to H/R Injury

**Figure 1:**
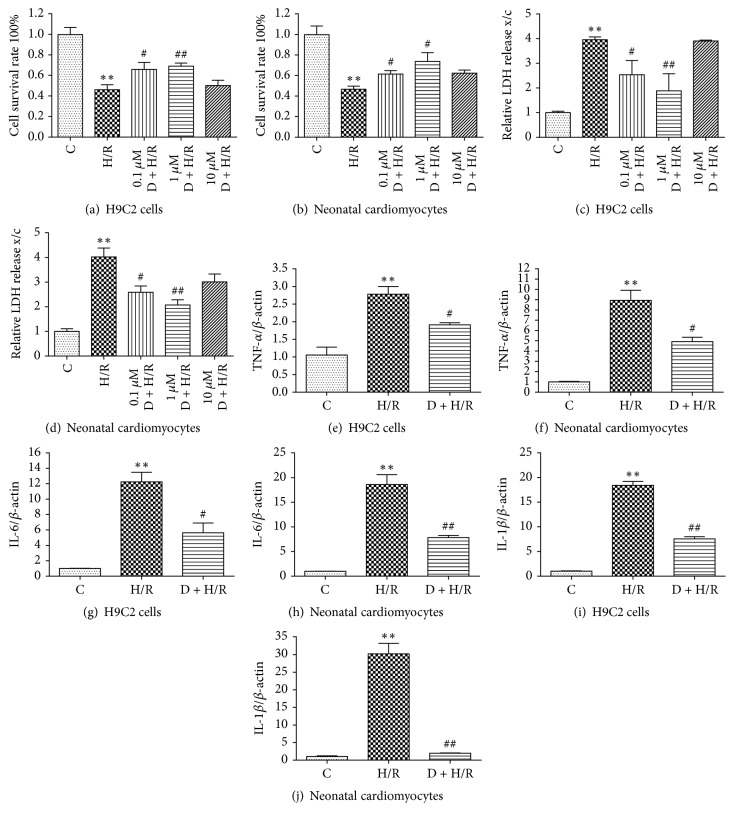
DEX pretreatment attenuates cell damage and inflammation in cardiomyocytes exposed to H/R. Viability of H9C2 cells and rat neonatal cardiomyocytes was measured by MTT (a, b) and LDH activity (c, d). Data are expressed as means ± SEM (*n* = 5 per group). IL-1*β*, TNF-*α*, and IL-6 expression was evaluated by quantitative RT-PCR (e–j). Data are expressed as means ± SEM (*n* = 3 per group). ^*∗∗*^*P* < 0.01 versus control group; ^#^*P* < 0.05, ^##^*P* < 0.01 versus H/R group. D: DEX.

After reperfusion, the survival rate of cardiomyocytes was markedly decreased in the H/R group compared to the control group. Cell survival rate was significantly improved by DEX pretreatment (Figures 1(a)-1(b)), with 1 *μ*M DEX offering the best protection. Consistent with the above finding, 1 *μ*M DEX pretreatment greatly diminished the H/R-induced increase in LDH levels (Figures 1(c)-1(d)). These data suggest that cell injury was reduced in the DEX pretreatment group. In addition, quantitative RT-PCR showed that TNF-*α*, IL-6, and IL-1*β* mRNA levels were significantly increased in the H/R group compared to the control, and DEX pretreatment partially blocked the increase in these inflammatory factors (Figures 1(e)–1(j)). Collectively, these findings suggest that DEX pretreatment substantially reduces I/R-triggered cell damage and inflammatory responses in cardiomyocytes.

### 3.2. DEX Pretreatment Suppresses TLR4-NF-*κ*B Signaling in Cardiomyocytes Exposed to H/R

**Figure 2:**
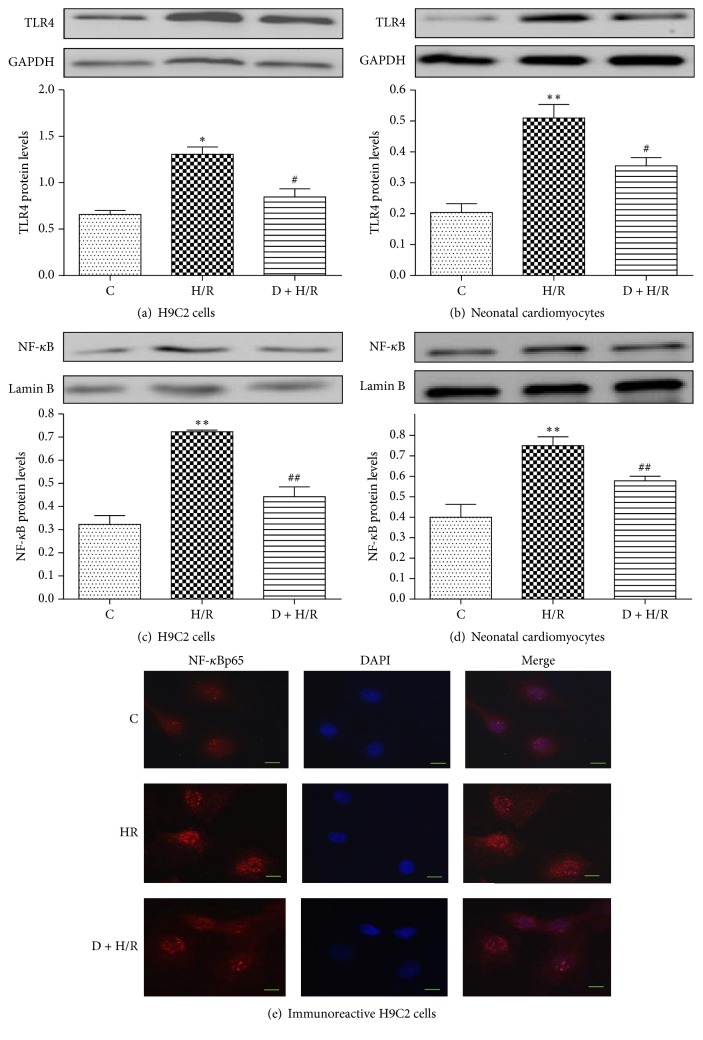
DEX pretreatment suppresses TLR4-NF-*κ*B signaling in cardiomyocytes exposed to H/R. TLR4 and nuclear NF-*κ*B p65 protein expression in H9C2 cells and rat neonatal cardiomyocytes was assessed by Western blot analysis (a–d). GAPDH and lamin B were used as a loading control for total and nuclear protein expression, respectively. Data are expressed as means ± SEM (*n* = 3 per group). ^*∗*^*P* < 0.05, ^*∗∗*^*P* < 0.01 versus control group; ^#^*P* < 0.05, ^##^*P* < 0.01 versus H/R group. Representative images showing the distributions of NF-*κ*B (red) in immunoreactive H9C2 cells (e). DAPI was used to stain nuclei. Magnification, 400x; scale bars = 10 *μ*m.

TLR4 and nuclear NF-*κ*B p65 expression in H9C2 cells and primary cardiomyocytes of the control and H/R groups were measured by Western blot analysis. As shown in Figures 2(a)–2(d), both TLR4 and nuclear NF-kB p65 protein levels increased significantly in the H/R group compared to the control group, and this effect was mitigated by DEX pretreatment. The above findings were further confirmed by IF assay in H9C2 cells. As shown in Figure 2(e), NF-*κ*B p65 nuclear translocation was promoted by H/R but was inhibited by DEX. These findings suggest that DEX pretreatment suppresses H/R-activation of TLR4-NF-kB signaling.

### 3.3. TLR4 Knock-Down by TLR4 siRNA Transfection Protects Cardiomyocytes against H/R Injury

**Figure 3:**
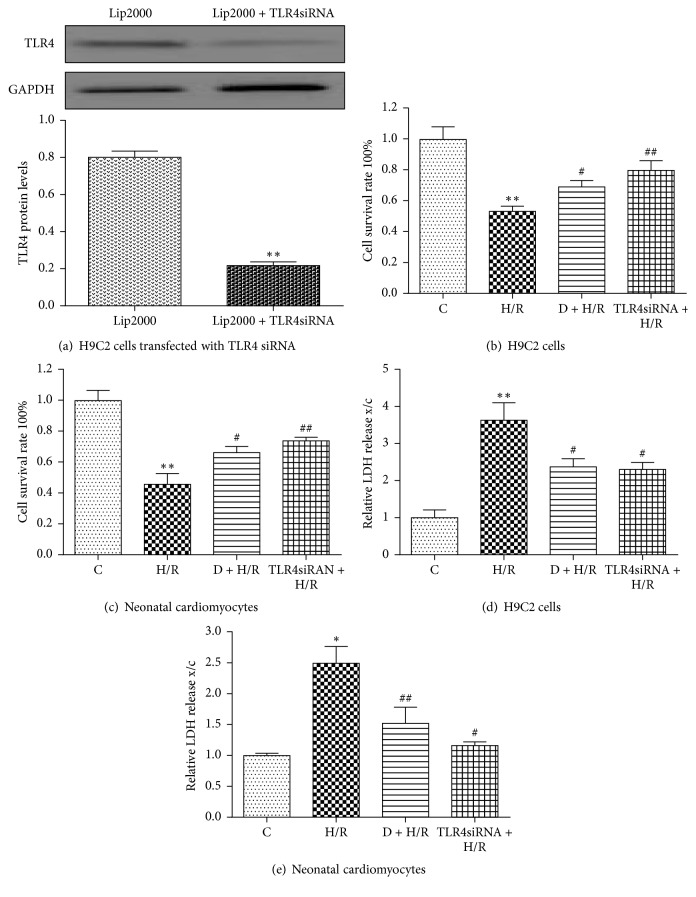
TLR4 knock-down by TLR4 siRNA transfection protects cardiomyocytes against H/R injury. TLR4 protein expression was detected in cells transfected with TLR4 siRNA for 24 h by Western blot analysis (a). GAPDH was used as a loading control. Data are expressed as means ± SEM (*n* = 3 per group). ^*∗∗*^*P* < 0.01 versus Lip2000 group. Viability of H9C2 cells and rat neonatal cardiomyocytes was measured by MTT (b, c) and LDH activity (d, e). Data are expressed as means ± SEM (*n* = 5 per group). ^*∗*^*P* < 0.05, ^*∗∗*^*P* < 0.01 versus control group; ^#^*P* < 0.05, ^##^*P* < 0.01 versus H/R group. D, DEX.

**Figure 4:**
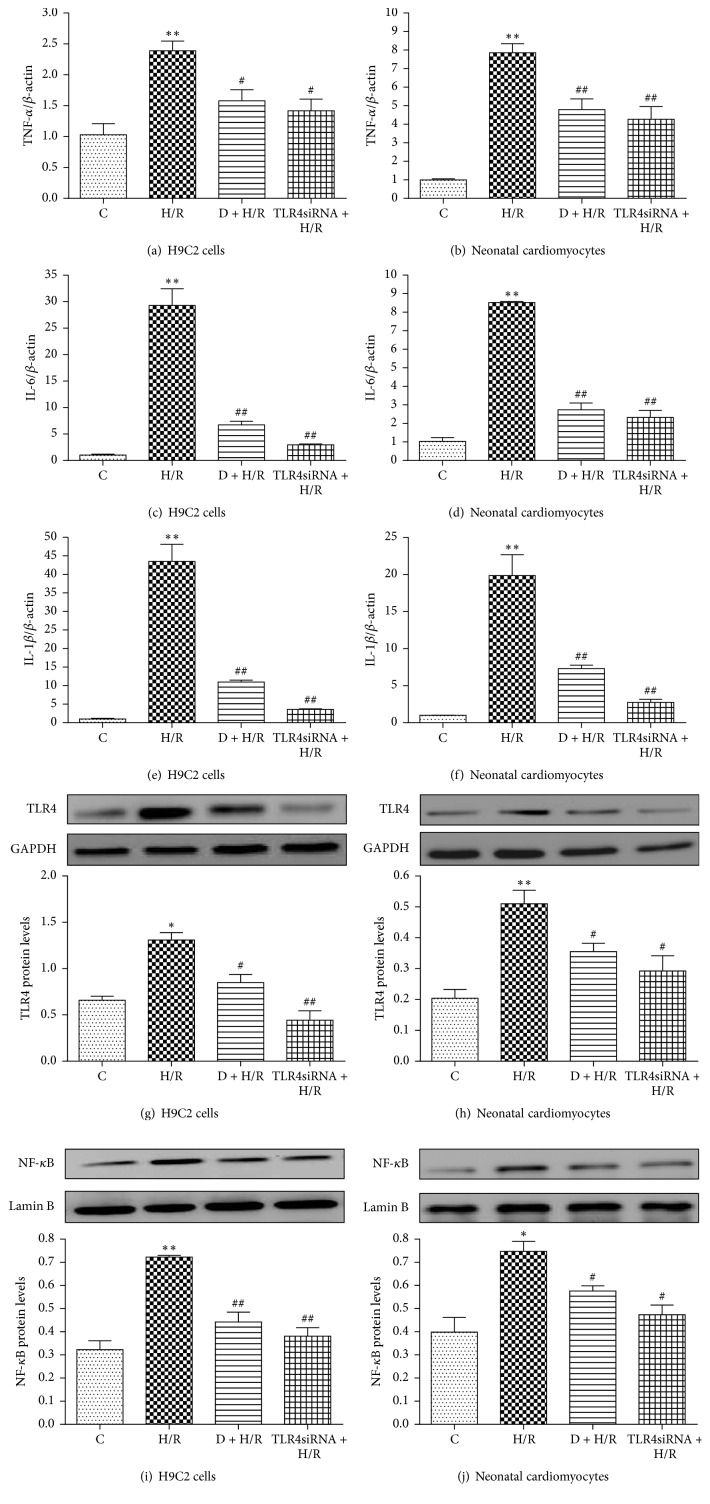
TLR4 knock-down by TLR4 siRNA transfection reduces TLR4 and nuclear NF-kB p65 levels in cardiomyocytes exposed to H/R. IL-1*β*, TNF-*α*, and IL-6 expression was measured by quantitative RT-PCR (a–f). Data are expressed as means ± SEM (*n* = 3 per group). Western blots were used to measure TLR4 and nuclear NF-*κ*B p65 protein expression in both H9C2 cells and neonatal cardiomyocytes (g–j). GAPDH and lamin B were used as a loading control for total and nuclear protein expression, respectively. Data are expressed as means ± SEM (*n* = 3 per group). ^*∗*^*P* < 0.05, ^*∗∗*^*P* < 0.01 versus control group; ^#^*P* < 0.05, ^##^*P* < 0.01 versus H/R group. D, DEX.

We next asked if TLR4 is involved in the DEX-mediated protective effect observed in H9C2 cells and cultured cardiomyocytes exposed to H/R. The efficiency of TLR4 gene knock-down was evaluated by Western blot analysis (Figure 3(a)). Compared to the H/R group, DEX pretreatment and TLR4 knock-down by TLR4 siRNA transfection both significantly increased cell survival rates and decreased LDH levels (Figures 3(b)–3(e)). TNF-*α*, IL-6, and IL-1*β* mRNA levels were also suppressed by either DEX pretreatment or TLR4 knock-down (Figures 4(a)–4(f)). In addition, TLR4 knock-down significantly reduced TLR4 and nuclear NF-*κ*B p65 protein levels (Figures 4(g)–4(j)). These findings suggest that TLR4 is involved in mediating H/R-induced cardiomyocyte injury.

### 3.4. Overexpression of TLR4 Reverses the Protective Effects of DEX

**Figure 5:**
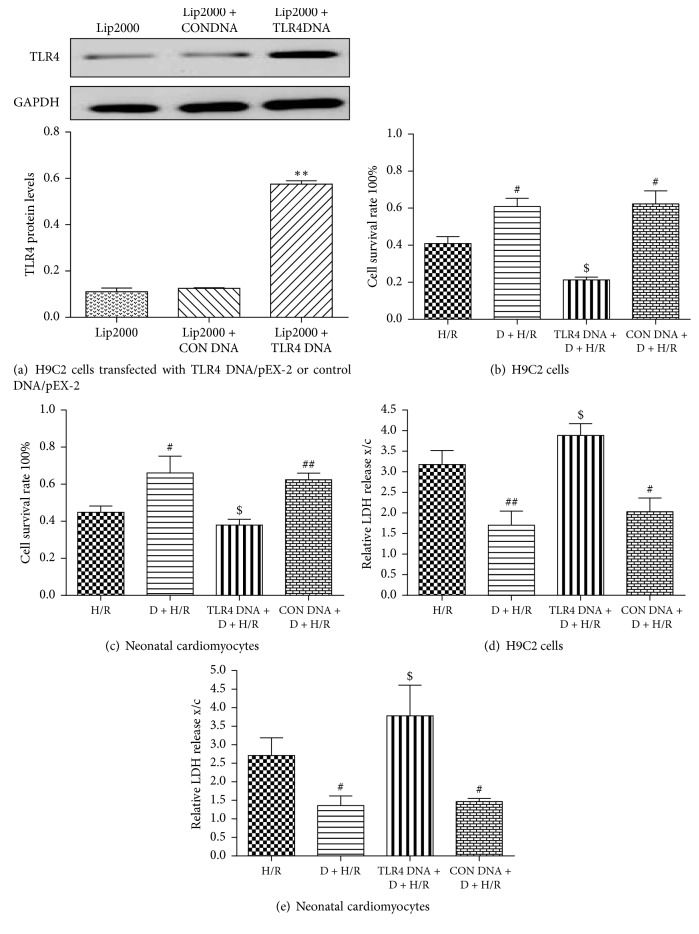
Overexpression of TLR4 reduces DEX-mediated protection against H/R-induced cell injury. TLR4 protein expression was detected in cells transfected with TLR4 DNA/pEX-2 or control DNA/pEX-2 for 24 h by Western blot analysis (a). GAPDH was used as a loading control. Data are expressed as means ± SEM (*n* = 3 per group). ^*∗∗*^*P* < 0.01 versus Lip2000 or Lip2000 + CONDNA group. Viability of H9C2 cells and rat neonatal cardiomyocytes were measured by MTT (b, c) and LDH activity (d, e). Data are expressed as means ± SEM (*n* = 5 per group). ^#^*P* < 0.05, ^##^*P* < 0.01 versus H/R group; ^$^*P* < 0.05. D, DEX.

**Figure 6:**
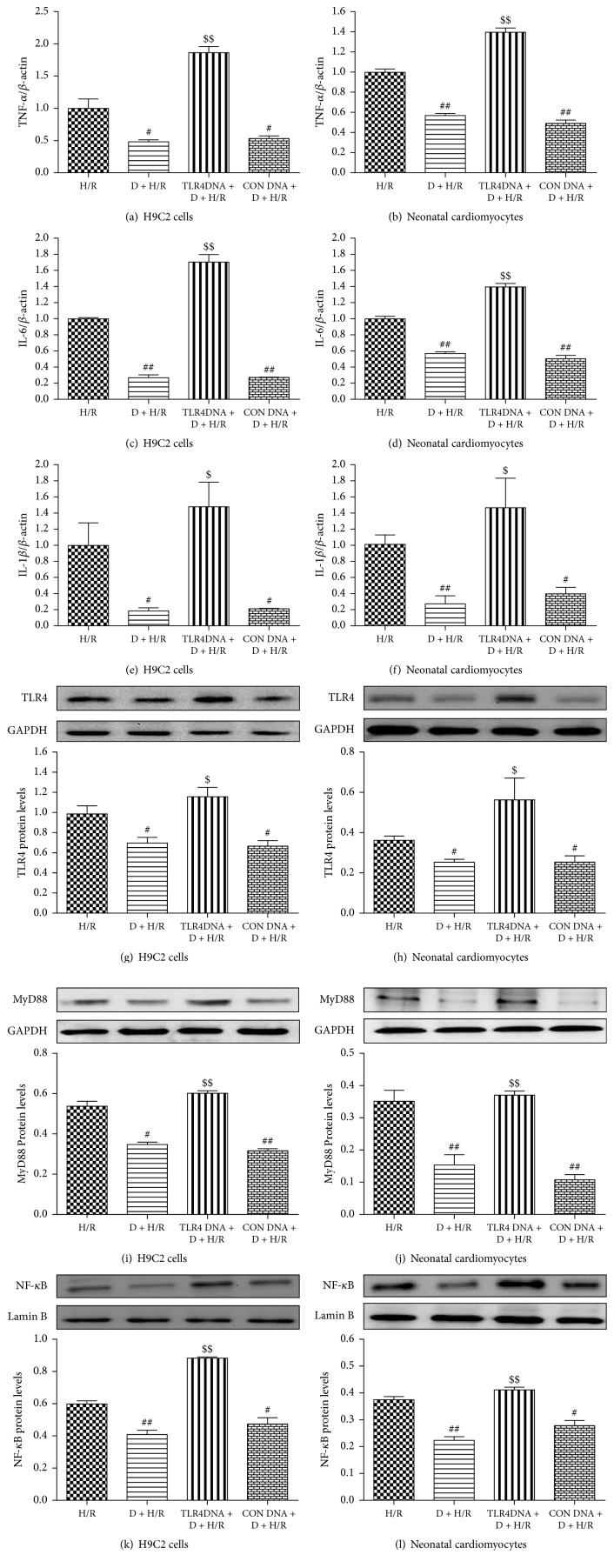
Overexpression of TLR4 reverses the inhibitory effects of DEX on TLR4, MyD88, and nuclear NF-kB p65 expression. IL-1*β*, TNF-*α*, and IL-6 expression was measured by quantitative RT-PCR (a–f). Data are expressed as means ± SEM (*n* = 3 per group). Western blot analysis was used to measure TLR4, MyD88, and nuclear NF-*κ*B p65 protein expression in both H9C2 cells and neonatal cardiomyocytes (g–l). GAPDH and lamin B were used as a loading control for total and nuclear protein expression, respectively. Data are expressed as means ± SEM (*n* = 3 per group). ^#^*P* < 0.05, ^##^*P* < 0.01 versus H/R group; ^$^*P* < 0.05, ^$$^*P* < 0.01 versus D + H/R group. D, DEX.

Next, we employed a gain-of-function approach to further examine the role of TLR4 in DEX-mediated cardioprotection. The efficiency of TLR4 gene overexpression was evaluated by Western blot analysis (Figure 5(a)). As shown above, DEX increased cell survival rate and decreased LDH activity and TNF-*α*, IL-6, and IL-1*β* expression in cells exposed to H/R. However, these beneficial effects were reversed by TLR4 overexpression, but not by transfection of control DNA (Figures 5(b)–5(e) and 6(a)–6(f)). Similarly, the DEX-mediated decrease in TLR4, MyD88, and nuclear NF-*κ*B p65 expression in H/R-exposed cells was significantly reversed by the transfected vector encoding TLR4, but not by the control DNA (Figures 6(g)–6(l)). Hence, we conclude that DEX-mediated cardioprotection against H/R involves TLR4 signaling.

## 4. Discussion

In the present study, we investigated the underlying mechanisms of DEX-mediated cardioprotection using* in vitro* H/R models. We demonstrated that DEX pretreatment protected cardiomyocytes against H/R injury, at least in part, by suppressing TLR4 and MyD88 expression and impeding NF-*κ*B translocation from the cytoplasm to the nucleus. Notably, we conducted this study using the H9C2 cardiac cell line as well as primary cultured rat neonatal cardiomyocytes to make the current results more reliable, as these two kinds of cells are used widely in the* in vitro* H/R model.

Hypoxia was generated using sodium hydrosulfite, a chemical oxygen scavenger, in the* in vitro* H/R models used in our study. Although hydrosulfite-mediated hypoxia is not equivalent to hypoxia caused by pathophysiologic vasoconstriction [23], it has still been used to successfully induce hypoxia in a variety of cells, including rat neonatal cardiomyocytes and H9C2 cells [18–21]. Most recently, we applied this model in primary neonatal rat cardiomyocytes and found that both preconditioning and postconditioning with DEX attenuated H/R injury at the cellular level [24].

Cell survival rate and LDH activity are generally used as indicators of cell injury. In the present study, we found that H/R caused severe cardiomyocyte membrane damage, decreased cell survival rate, and increased LDH activity. However, these injury-related effects were ameliorated by DEX pretreatment. Moreover, we showed that 1 *μ*M DEX offered the best protection. In addition, we found that H/R increased the levels of proinflammatory cytokines (TNF-*α*, IL-1*β*, and IL-6) in cardiomyocytes, all of which were previously shown to be directly involved in the progression of myocardial I/R injury, myocardial dysfunction, vascular wall remodeling, heart failure, and cardiac hypertrophy [25, 26]. In line with our previous results [14, 15], the present study confirmed that DEX protects cardiomyocytes against H/R injury via an anti-inflammatory response.

TLRs act as sentinels of tissue damage and mediators of inflammatory responses following pathogen detection [27]. TLR4 is primarily expressed in myocardial cells [3] and can be activated by either exogenous or endogenous ligands to induce downstream signals that lead to cytokine and chemokine production and inflammatory responses [28–31]. The TLR4-NF-*κ*B axis is a key signaling pathway in myocardial I/R injury [32]. NF-*κ*B stays in an inactive state in the cytoplasm when bound to the inhibitory I*κ*B subunit [33]. However, in response to external stimuli, the I*κ*B subunit is phosphorylated, resulting in the release and translocation of NF-*κ*B to the nucleus, where it triggers the transcription of downstream target genes involved in the inflammatory response [34, 35]. NF-*κ*B also promotes production of major inflammatory mediators including TNF-*α*, IL-1*β*, and IL-6, which have been implicated in myocardial apoptosis and death [4, 36, 37]. In addition, it is well known that TLR4 signaling activated by MyD88 and TIRAP mediates rapid activation of NF-kB and MAPKs, which in turn induces MyD88-dependent activation of cytokines, such as TNF-*α* and IL-1*β* or TRAM and TRIF, to increase IFN-*β* production [38–40]. Therefore, we measured MyD88 levels downstream of TLR4 receptor activation in this study.

It has been shown that DEX acts as an anti-inflammatory agent and provides cardioprotection by increasing expression of cell survival proteins, improving postischemic ventricular recovery, and reducing myocardial infarct size and cardiomyocyte apoptosis [11, 41–43]. Mechanistically, it was previously reported that the antiapoptotic and anti-inflammatory effects of DEX in I/R injury were related to phosphoinositide 3-kinase- (PI3K-) AKT and extracellular regulated kinase 1/2 (ERK1/2) signaling pathways [44]. Previously, Ibacache et al. reported that DEX preconditioning produced cardioprotection against I/R injury by the activation of prosurvival kinases after cardiac *α*2-adrenergic receptor stimulation [11]. In our recent* in vivo* and* ex vivo* experiments, we also verified that the addition of yohimbine, the selective *α*2-adrenergic receptor antagonist, greatly attenuated DEX-induced cardioprotection [14, 15]. Besides, DEX-induced cardioprotection may be attributed to the downregulation of the HMGB1-TLR4-MyD88-NF-кB signaling pathway [14]. In this study, we used TLR4 knock-down by TLR4 siRNA transfection and overexpression by TLR4 DNA transfection* in vitro* approaches to explore the mechanisms underlying DEX-mediated cardioprotection. We demonstrated that DEX has anti-inflammatory activity and that TLR4-MyD88-NF-*κ*B signaling is involved in the DEX-mediated cardioprotection against H/R injury.

Some limitations of the present study need to be acknowledged. For example, we used* in vitro* cardiomyocyte H/R models to mimic I/R injury in rats to explore the molecular basis underlying DEX's cardioprotection. Thus, the differences between* in vitro* and* in vivo* models need to be taken into consideration when interpreting the data. In addition, we used the H9C2 cell line and rat neonatal cardiomyocytes instead of adult cardiomyocytes, so the physiological benefits offered by DEX will need to be further investigated in adult rat cardiomyocytes, or even human cardiomyocytes, to more accurately evaluate the potential clinical benefits of DEX treatment. Further study of the precise mechanisms responsible for the cardioprotective effects of DEX is currently under way.

## 5. Conclusions

We demonstrated that DEX preconditioning offers cardioprotection, at least in part, by TLR4 suppression via TLR4-MyD88-NF-*κ*B signaling. The perioperative use of DEX may be a potentially potent therapeutic strategy for high-risk patients undergoing cardiac surgery.
